# A biohybrid nanovesicle hijacks inflammatory chemotaxis to deliver colchicine for myocardial infarction therapy

**DOI:** 10.3389/fbioe.2026.1751640

**Published:** 2026-04-02

**Authors:** Xiangyong Hu, Liping Du, Hongju Xiang, Yuyu Li, Zhixiong Liao, Jiaqi Yu

**Affiliations:** 1 Department of Cardiovascular Medicine, Xiangxi Tujia and Miao Autonomous Prefecture People’s Hospital, The First Affiliated Hospital of Jishou University, Jishou, Hunan, China; 2 Medical College of Jishou University, Jishou, Hunan, China; 3 Department of Cardiology, The First Affiliated Hospital of Wenzhou Medical University, Wenzhou, Zhejiang, China; 4 Department of Cardiovascular Medicine, Zhangjiajie People’s Hospital, Jishou University, Jishou, Hunan, China; 5 Beijing Institute of Heart, Lung and Blood Vessel Disease, Beijing Anzhen Hospital Affiliated to Capital Medical University, Beijing, China

**Keywords:** biomimetic nanovesicles, colchicine, inflammation, myocardial infarction, neutrophil

## Abstract

**Introduction:**

Myocardial infarction (MI) triggers an excessive inflammatory response that drives adverse cardiac remodeling. Although colchicine has shown clinical promise, its narrow therapeutic window and lack of target specificity limit its efficacy. To address these limitations, we developed a biohybrid nanovesicle (MM‐LP@COL) that encapsulates colchicine, aiming to leverage natural inflammatory tropism for site-specific drug delivery.

**Methods:**

MM‐LP@COL nanovesicles were fabricated by fusing macrophage membranes with liposomes. Physicochemical characterization was performed using DLS, TEM, and FRET analysis. Cytokine‐scavenging capacity was evaluated *in vitro* by ELISA. In a murine MI model, mice were treated with PBS, free colchicine, LP@COL, or MM‐LP@COL. Immune cell populations in blood, bone marrow, and spleen were analyzed by high‐dimensional flow cytometry (UMAP). Cardiac function and tissue remodeling were assessed by echocardiography and histology.

**Results:**

MM‐LP@COL formed uniform spherical nanovesicles (∼200 nm) and demonstrated potent, dose‐dependent neutralization of multiple pro‐inflammatory cytokines *in vitro*, along with reduced circulating cytokine levels in MI mice. High‐dimensional cytometric analysis revealed that treatment significantly reduced neutrophil infiltration across key immune organs, including blood, bone marrow, and spleen. These effects translated into improved left ventricular function, reduced myocardial inflammation, and attenuated cardiomyocyte hypertrophy.

**Discussion:**

This study demonstrates that the macrophage-inspired nanovesicle system constitutes a potent and targeted combinatorial therapy for post-infarction inflammation and repair.

## Introduction

Myocardial infarction (MI) triggers a profound and dysregulated inflammatory response that is a major contributor to adverse cardiac remodeling and progression to heart failure (Frangogiannis, 2014; Wang et al., 2019; Yu et al., 2024). While inflammation is initially a reparative process, its excessive and prolonged activation, characterized by a massive influx of neutrophils and the uncontrolled release of pro-inflammatory cytokines, leads to further cardiomyocyte death and matrix degradation (Fung et al., 2016; Zhang et al., 2016; Jung et al., 2017; Li et al., 2024). Consequently, modulating this maladaptive immune response has emerged as a critical therapeutic frontier, alongside revascularization strategies (DeBerge et al., 2017).

Colchicine, a classic anti-inflammatory medication, has recently gained significant attention in cardiology (Adler et al., 2015; Li et al., 2021; Li et al., 2022). Groundbreaking clinical trials have demonstrated that low-dose oral colchicine significantly reduces adverse cardiovascular events in patients post-MI (Bouabdallaoui et al., 2020; Nidorf et al., 2020), attributed to its potent inhibitory effects on neutrophil activation (Van Wagoner, 2011; Cao et al., 2016) and the NLRP3 inflammasome (Misawa et al., 2013; Robertson et al., 2016; Tardif et al., 2019; Sun et al., 2022). However, its clinical utility is constrained by a narrow therapeutic index and dose-dependent systemic toxicities, notably gastrointestinal adverse effects and myelosuppression (Finkelstein et al., 2010; van Echteld et al., 2014; Slobodnick et al., 2018). Furthermore, lack of inherent target specificity for the infarcted myocardium compromises its local efficacy and necessitates careful dose management.

To overcome these limitations, we engineered a biohybrid nanovesicle designed for targeted and synergistic anti-inflammatory therapy (Gao et al., 2025). This system, termed MM-LP@COL, was fabricated by fusing macrophage membranes (MM) with synthetic liposomes (LP) to encapsulate colchicine. This innovative design integrates two distinct anti-inflammatory modalities. First, the macrophage membrane shell, which naturally expresses a repertoire of chemokine and cytokine receptors (Li et al., 2022; Li et al., 2023), acts as a decoy to bind and neutralize a broad spectrum of pro-inflammatory mediators in the circulation and the infarct microenvironment (Veillette and Chen, 2018). This intrinsic “cytokine-neutralizing” property provides an immediate, drug-independent therapeutic effect. In addition, this biomimetic coating enables the nanovesicles to inherit the innate tropism of macrophages for inflammatory sites, thereby achieving targeted delivery of the encapsulated colchicine directly to the injured heart.

Here, we hypothesize that MM-LP@COL would orchestrate a comprehensive anti-inflammatory response through this dual mechanism: the decoy effect of the macrophage membrane and the targeted action of colchicine. This synergistic approach is anticipated to more effectively mitigate the systemic and local inflammatory storm, thereby disrupting the deleterious cascade of neutrophil recruitment from the bone marrow and reducing infiltration into secondary lymphoid organs and the infarcted tissue. Ultimately, this strategy aims to achieve superior cardio protection, significantly attenuate adverse remodeling, and improve long-term cardiac function, presenting a promising nanotherapeutic strategy for post-infarction healing.

## Results and discussion

### Successful fabrication and characterization of MM-LP@COL nanovesicles

To develop a targeted anti-inflammatory nanoplatform for myocardial infarction, biomimetic nanovesicles (MM-LP) were fabricated by fusing macrophage membranes (MM) with liposomes (LP). The fabrication process and key physicochemical properties of the resulting nanovesicles were systematically characterized.

**FIGURE 1:**
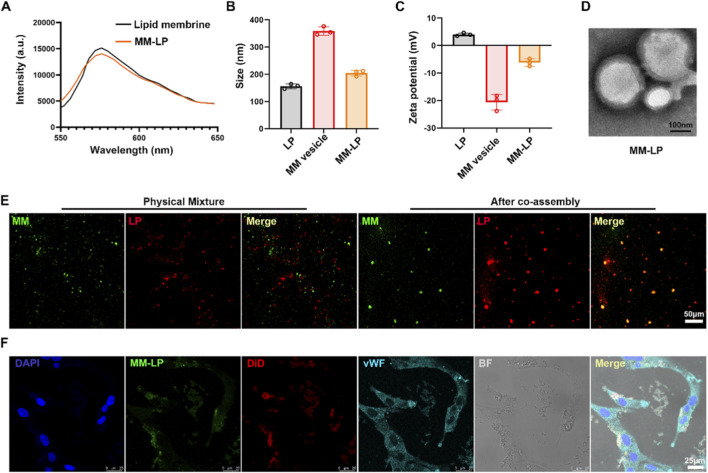
Preparation and characterization of MM-LP@COL nanovesicles. **(A)** FRET analysis validating the fusion between the DiO/DiI-labeled lipid membrane and the macrophage membrane. The recorded fluorescence intensity profile confirms successful integration. **(B)** Hydrodynamic diameter distribution of the nanovesicles measured by dynamic light scattering (DLS). Data are presented as mean ± standard deviation (n = 3). **(C)** Zeta potential distribution of the synthesized nanovesicles. Data are presented as mean ± standard deviation (n = 3). **(D)** Representative transmission electron microscopy (TEM) image revealing the morphology of MM-LP nanovesicles. Scale bar, 100 nm. **(E)** Confocal laser scanning microscopy (CLSM) image of MM-LP nanoparticles. A simple physical mixture of the two components served as the control. Scale bar, 50 μm. **(F)** Cellular uptake analysis. CLSM images of endothelial cells (ECs) following a 4-h incubation with MM-LP@DiD NPs. The MM-LP component was labeled with DiO (green), and the COL was replaced with DiD (red). Cells were pre-stimulated with TNF-α to induce expression of von Willebrand factor (vWF, aqua). Nuclei were counterstained with DAPI (blue). The composite image is an overlay of the bright field (BF) and all fluorescence channels. Scale bar, 25 μm.

First, the successful fusion between the macrophage membranes and the synthetic liposomes was verified using a Förster resonance energy transfer (FRET) assay. The liposomal membrane was dual-labeled with the donor DiO and the acceptor DiI. A marked decrease in the DiI fluorescence intensity at 565 nm was observed following the introduction of unlabeled macrophage membranes (Figure 1A). This quenching of acceptor emission confirmed the integration of macrophage membranes into the lipid bilayers, which increased the separation distance between the FRET pair and thus validated successful membrane fusion.

The physical characteristics of the nanovesicles were subsequently analyzed. Dynamic light scattering (DLS) revealed that the MM-LP nanovesicles exhibited a uniform size distribution with a hydrodynamic diameter of approximately 200 nm (Figure 1B). The surface charge, as determined by zeta potential measurement, was moderately negative for the MM-LP formulation, a value intermediate between those of plain liposomes and pure macrophage membrane vesicles, providing further evidence of their hybrid nature (Figure 1C). Morphological examination by transmission electron microscopy (TEM) confirmed that the MM-LP nanovesicles were spherical and unilamellar, with a size consistent with the DLS measurements (Figure 1D).

To visually confirm the colocalization of the macrophage membrane shell and the liposomal core in the resulting nanostructure, confocal laser scanning microscopy (CLSM) was performed. The macrophage membrane and liposomal component were specifically labeled with two distinct fluorescent dyes. A pronounced overlap of the two fluorescence signals was observed in the MM-LP nanovesicles, in clear contrast to the signal distribution in a simple physical mixture of the components, thus corroborating the formation of an integrated biohybrid structure (Figure 1E).

Finally, cellular interactions of the nanovesicles were evaluated *in vitro*. TNF-α-prestimulated endothelial cells (ECs) were incubated with MM-LP@DiD nanovesicles containing DiD-labeled liposomal cores. CLSM imaging confirmed efficient cellular uptake by activated ECs, as shown by distinct intracellular red fluorescence (Figure 1F). This finding demonstrates that the macrophage membrane coating preserves the innate targeting capability toward inflammatory endothelium, an essential requirement for site-specific drug delivery to inflamed tissues. To further investigate whether the MM-LP nanovesicles inherited critical physiological membrane proteins from their macrophage source, Western blotting was performed. Analysis confirmed the retention of key proteins involved in targeting activated endothelium, such as adhesion molecule receptors, and the immune checkpoint protein crucial for immune evasion. The results indicated that MM-LP nanovesicles retained these proteins similarly to pure macrophage membrane vesicles (Supplementary Figure 1). In addition, the drug loading efficiency (LE) of colchicine in the nanovesicles was determined to be 3.76 ± 0.14%, and the encapsulation efficiency (EE) was 38.63 ± 0.15%. In summary, MM-LP@COL nanovesicles were successfully fabricated with desirable nano-properties, and their core design principle, a fused macrophage membrane-liposome structure capable of efficient cellular uptake was validated.

### MM-LP nanovesicles effectively neutralize inflammatory cytokines

Cardiovascular diseases, such as atherosclerosis and myocardial infarction, are commonly underpinned by severe inflammatory responses (Lusis, 2000; Frangogiannis et al., 2002; Nahrendorf et al., 2010). Within the MM-LP nanomedicine delivery system, owing to its inheritance of inflammatory receptors present on macrophage membranes, such as TLR4, CD126, and CD119, MM-LP exhibits a potent capacity for adsorbing inflammatory mediators (Bai et al., 2025). Thus, the inherent cytokine-neutralizing capacity of the MM-LP nanovesicles, conferred by the macrophage membrane shell, was systematically evaluated both *in vitro* and *in vivo*.

**FIGURE 2:**
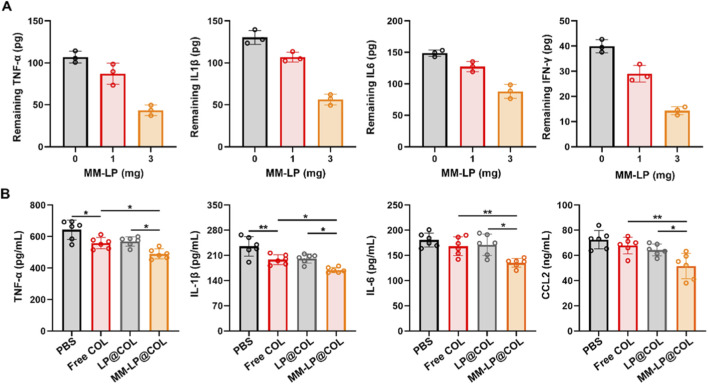
Anti-inflammatory efficacy of MM-LP@COL nanovesicles. **(A)** *In vitro* cytokine neutralization by MM-LP nanovesicles. Neutralization capacity against TNF-α, IL-1β, IL-6, and IFN-γ was assessed following incubation with increasing concentrations of MM-LP nanovesicles (0, 1, and 3 mg/mL). Data are presented as mean ± SD (n = 3). **(B)** Systemic anti-inflammatory effects in a murine myocardial infarction model. Serum levels of TNF-α, IL-1β, IL-6, and CCL2 were measured by ELISA after treatment with PBS, Free COL, LP@COL, or MM-LP@COL nanovesicles (n = 6 per group). *p < 0.05, and **p < 0.01, data are presented as the mean ± SD and analyzed by using one-way ANOVA, Tukey’s multiple comparisons test.

Initially, the decoy function of the nanovesicles was quantified *in vitro*. MM-LP nanovesicles at varying concentrations were incubated with predetermined amounts of key pro-inflammatory cytokines (TNF-α, IL-1β, IL-6, IFN-γ). After the removal of nanovesicles by centrifugation, the residual cytokine concentrations in the supernatant were measured via ELISA. As demonstrated in Figure 2A, a dose-dependent neutralization of all tested cytokines was observed. The neutralizing effect was significant at a concentration of 1 mg and was substantially enhanced at 3 mg, confirming the broad-spectrum cytokine-scavenging capacity of the nanovesicles.

The efficacy of this neutralization was further validated in a murine MI model. Serum levels of TNF-α, IL-1β, IL-6, and CCL2 were measured following treatment with different formulations. As shown in Figure 2B, the MM-LP@COL treatment group exhibited the most robust suppression of circulating cytokine levels compared to the PBS, Free COL, and LP@COL groups. This result indicates that the MM-LP platform effectively functions as a cytokine scavenger *in vivo*, significantly attenuating the systemic inflammatory storm post-MI.

### MM-LP@COL nanovesicles specifically reduce blood neutrophil levels in post-myocardial infarction mice

**FIGURE 3:**
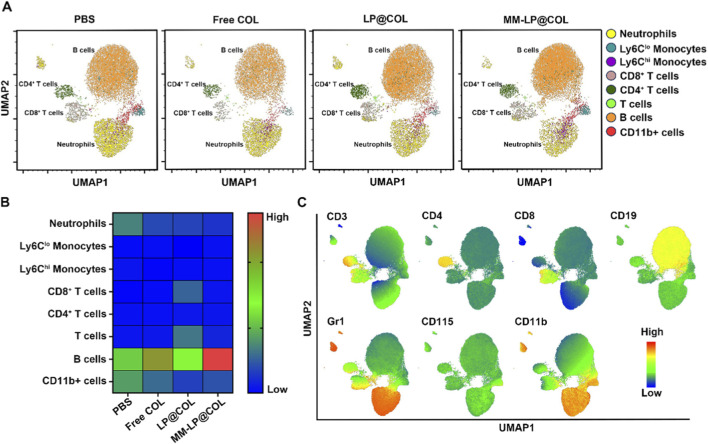
MM-LP@COL nanovesicles reduce neutrophil levels in the blood of post-myocardial infarction mice. **(A)** UMAP analysis of immune cells in peripheral blood. Representative UMAP plots are shown for myocardial infarction (MI) model mice treated with PBS, Free COL, LP@COL, or MM-LP@COL nanovesicles (n = 3 per group). **(B)** Heatmap depicting the relative quantification of distinct immune cell populations across the different treatment groups. **(C)** UMAP plots illustrating the expression patterns of key immune cell markers within each identified metacluster.

To evaluate the systemic immunomodulatory effects of the nanovesicles, we performed high-dimensional flow cytometric analysis of peripheral blood samples from myocardial infarction model mice treated with PBS, Free COL, LP@COL, or MM-LP@COL. Single-cell suspensions from peripheral blood were analyzed using a comprehensive multicolor flow cytometry panel containing myeloid cell markers (CD11b, GR1) and lymphocyte markers (CD3, CD4, CD8). The high-dimensional data were processed using the UMAP algorithm, with unsupervised clustering enabling identification of distinct immune cell subsets in an entirely unbiased manner (Figure 3A). Heatmap visualization illustrated the relative abundance of different immune cell populations across treatment groups. Quantitative assessment specifically revealed that neutrophil levels in peripheral blood were most significantly reduced in the MM-LP@COL group compared to both Free COL and LP@COL treatments (Figure 3B). Further UMAP analysis visualized the expression profiles of characteristic markers for each metacluster, including CD11b and GR1 for neutrophils, CD115 for monocytes, and CD3, CD4, CD8 for T cell subsets, confirming comprehensive immunophenotyping of peripheral immune cells (Figure 3C). These findings demonstrate that MM-LP@COL nanovesicles effectively reduce neutrophil levels specifically in the blood circulation following myocardial infarction, showing superior efficacy to both free drug and non-targeted nanocarrier approaches.

### MM-LP@COL nanovesicles inhibit bone marrow neutrophil mobilization after myocardial infarction

**FIGURE 4:**
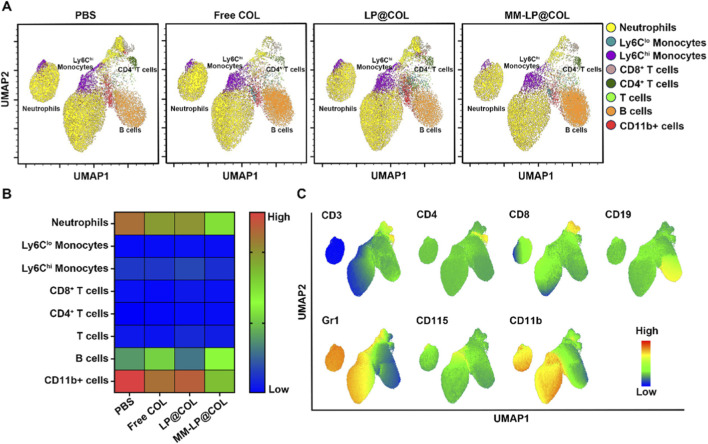
MM-LP@COL nanovesicles attenuate neutrophil mobilization from the bone marrow following myocardial infarction. **(A)** UMAP analysis of bone marrow immune cells from MI mice treated with PBS, Free COL, LP@COL, or MM-LP@COL nanovesicles (n = 3 per group). **(B)** Heatmap showing the relative abundance of immune cell populations in bone marrow across treatment groups. **(C)** UMAP plots displaying expression patterns of characteristic surface markers within each metacluster.

To investigate the origin and regulation of neutrophil infiltration, we extended our high-dimensional flow cytometric analysis to bone marrow samples from myocardial infarction model mice treated with PBS, Free COL, LP@COL, or MM-LP@COL. Single-cell suspensions from bone marrow were analyzed using the same multicolor flow cytometry panel established. The UMAP algorithm visualization of bone marrow-derived immune cells revealed distinct compositional changes among treatment groups (Figure 4A). Heatmap analysis of the relative abundance of immune cell populations in bone marrow demonstrated that MM-LP@COL treatment specifically and significantly reduced the neutrophil proportion compared to other treatment groups (Figure 4B). The observed contraction of the bone marrow neutrophil pool provides a mechanistic basis for the decreased peripheral neutrophil levels noted in earlier experiments. Further UMAP analysis visualized the expression profiles of characteristic markers for each metacluster (Figure 4C). These results indicate that MM-LP@COL nanovesicles effectively suppress neutrophil mobilization at its source in the bone marrow, representing a crucial mechanism through which this targeted nanotherapy attenuates systemic neutrophilic inflammation following myocardial infarction.

### MM-LP@COL nanovesicles attenuate splenic neutrophil accumulation following myocardial infarction

**FIGURE 5:**
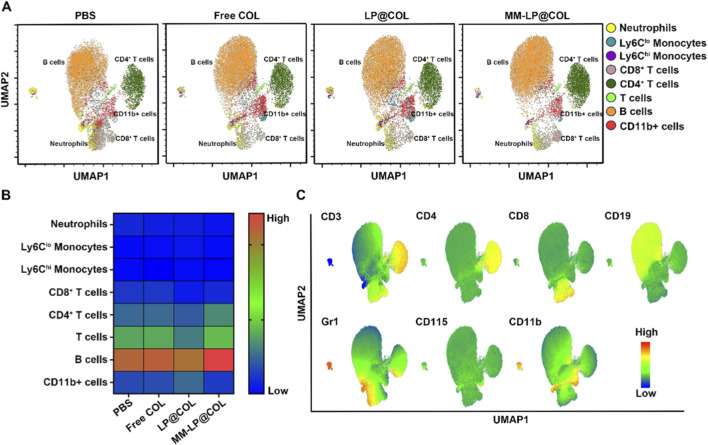
MM-LP@COL nanovesicles mitigate neutrophil accumulation in the spleen after myocardial infarction. **(A)** UMAP analysis of splenic immune cells. Representative UMAP plots are shown for MI mice treated with PBS, Free COL, LP@COL, or MM-LP@COL nanovesicles (n = 3 per group). **(B)** Heatmap displaying the relative proportions of various immune cell types in the spleen across different treatment groups. **(C)** UMAP plots demonstrating the expression patterns of specific immune cell markers within each metacluster.

High-dimensional flow cytometric analysis of splenic samples was employed to further assess the impact of nanovesicles on secondary lymphoid organs in a murine model of myocardial infarction (MI) under various treatment regimens. UMAP visualization of splenic immune cells revealed substantial alterations in cellular composition among the treatment groups (Figure 5A). The overall distribution pattern of immune cells in the MM-LP@COL group displayed distinct differences from both the PBS control and other treatment groups. Heatmap analysis further demonstrated that MM-LP@COL treatment led to a reduction in splenic neutrophil proportions compared to Free COL and LP@COL treatments (Figure 5B). This decrease in splenic neutrophil accumulation suggests that MM-LP@COL nanovesicles effectively inhibit neutrophil trafficking to secondary lymphoid organs post-myocardial infarction. Comprehensive UMAP mapping of marker expression patterns within each metacluster confirmed the identity of various immune cell populations, including neutrophils, monocytes, and lymphocyte subsets, thereby validating the cellular classification (Figure 5C). Collectively, these results indicate that MM-LP@COL nanovesicles effectively limit neutrophil infiltration not only in circulation and bone marrow but also in secondary lymphoid organs, demonstrating their comprehensive suppressive effect on the neutrophil-mediated inflammatory cascade after myocardial infarction.

### MM-LP@COL nanovesicles confer long-term therapeutic benefits after myocardial infarction

**FIGURE 6:**
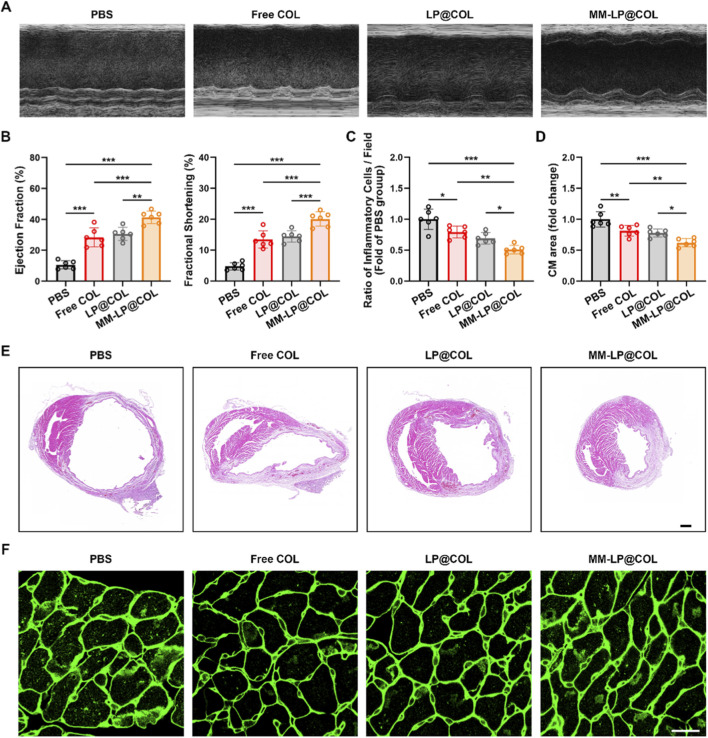
MM-LP@COL nanovesicles confer long-term therapeutic benefits after myocardial infarction. **(A)** Representative echocardiographic images of myocardial infarction (MI) mice treated with PBS, Free COL, LP@COL, or MM-LP@COL. **(B)** Quantitative analysis of cardiac function parameters: left ventricular ejection fraction (EF, left) and fractional shortening (FS, right). Data are presented as mean ± SD (n = 6). **(C)** Quantification of inflammatory cell infiltration in cardiac tissue based on H&E-stained sections across treatment groups. Data are presented as mean ± SD (n = 6). **(D)** Measurement of cardiomyocyte cross-sectional area from WGA-stained sections. Data are presented as mean ± SD (n = 6). **(E)** Representative photomicrographs of H&E-stained myocardial tissue. Scale bar, 500 μm. **(F)** Representative fluorescent images of WGA-stained myocardial tissue, outlining cardiomyocyte membranes. Scale bar, 20 μm. *p < 0.05, **p < 0.01, and ***p < 0.001, data are presented as the mean ± SD and analyzed by using one-way ANOVA, Tukey’s multiple comparisons test.

The long-term therapeutic efficacy of MM-LP@COL was evaluated through comprehensive assessment of cardiac function and structural remodeling. To validate the inflammatory tropism conferred by the macrophage membrane coating and distinguish it from passive effects, we directly compared the cardiac accumulation of targeted versus non-targeted nanovesicles in a murine MI model. As shown in Supplementary Figure 2, *ex vivo* fluorescence imaging of heart tissues harvested 3 days post-injection revealed a significantly higher fluorescence signal in the MM-LP@DiD group compared to the LP@DiD group. This enhanced and specific accumulation at the infarcted heart at this early time point strongly supports an active targeting mechanism mediated by the macrophage membrane coating, which operates beyond passive enhanced permeability and retention (EPR) effects. Representative echocardiographic images demonstrated improved cardiac morphology in the MM-LP@COL treatment group compared to other formulations (Figure 6A). Quantitative functional analysis confirmed that MM-LP@COL treatment significantly improved left ventricular ejection fraction (EF) and fractional shortening (FS) compared to PBS, Free COL, and LP@COL groups (Figure 6B), indicating substantial preservation of cardiac contractile function.

Histopathological assessment revealed that MM-LP@COL treatment markedly reduced inflammatory cell infiltration in myocardial tissue compared to other treatment groups (Figure 6C). Moreover, measurement of cardiomyocyte cross-sectional area indicated that MM-LP@COL significantly suppressed pathological hypertrophy (Figure 6D). Representative H&E-stained myocardial sections visually supported these findings, showing attenuated inflammatory infiltration and better-maintained tissue architecture in the MM-LP@COL group (Figure 6E). Similarly, WGA-stained sections provided clear evidence of the protective effect of MM-LP@COL against cardiomyocyte enlargement (Figure 6F). Together, these results indicate that MM-LP@COL nanovesicles confer sustained cardio protection by improving cardiac function, mitigating inflammation, and restraining maladaptive remodeling after myocardial infarction.

The superior therapeutic outcomes observed with MM-LP@COL can be attributed to a synergistic mechanism orchestrated by its unique biohybrid design. Beyond functioning as a simple drug carrier coating, the macrophage membrane shell acts as a multifaceted therapeutic component. Its well-documented capacity to neutralize a broad spectrum of pro-inflammatory cytokines (TNF-α, IL-1β, IL-6, CCL2) provides an immediate, drug-independent anti-inflammatory effect, mitigating the systemic “cytokine storm” post-MI. More importantly, this membrane is not inert; it is endowed with active targeting capabilities. The inherited repertoire of chemokine receptors and adhesion molecules from the source macrophage membrane, including receptors such as CCR2 and CXCRs, enables MM-LP@COL to actively recognize and bind to adhesion molecules and chemokines, such as ICAM-1, VCAM-1, and MCP-1, that are highly upregulated on activated endothelium in the infarcted and border zones. This biological tropism facilitates the active navigation and selective accumulation of nanovesicles at the site of inflammation, far surpassing the passive accumulation typically observed with conventional liposomes such as LP@COL.

This active targeting synergizes profoundly with the encapsulated colchicine. The membrane-mediated homing ensures a high local concentration of colchicine precisely where it is most needed, within the inflamed myocardial tissue. Once internalized by infiltrating immune cells and resident cardiac cells, colchicine exerts its potent intracellular effects, primarily by inhibiting microtubule polymerization and suppressing the NLRP3 inflammasome activation. This targeted delivery likely explains the markedly enhanced efficacy in curbing neutrophil mobilization from the bone marrow, their subsequent infiltration into secondary lymphoid organs like the spleen, and their final recruitment into the heart, as evidenced in Figures 3–5. The decoy effect of the membrane reduces the chemotactic signals in the circulation, while the targeted delivery of colchicine disrupts the cellular response to any remaining signals at the target site. The combined action of extracellular cytokine clearance by the membrane and intracellular anti-inflammatory modulation by colchicine synergistically disrupts the neutrophilic inflammatory cascade. This mechanism underlies the significant preservation of cardiac function, attenuation of pathological remodeling, and improved long-term recovery demonstrated in this study.

The promising preclinical efficacy of MM-LP@COL necessitates a candid discussion of the translational challenges and future steps toward clinical application. First, the scalability and batch-to-batch reproducibility of macrophage membrane isolation and subsequent fusion with synthetic liposomes must be rigorously addressed. While our laboratory-scale co-extrusion method yielded consistent nanovesicles, future work will require process optimization for larger-scale production, potentially exploring scalable techniques like microfluidic mixing. Second, the potential immunogenicity of membranes derived from allogeneic Raw 264.7 macrophage cells warrants careful evaluation in immunocompetent models over extended periods. Although macrophage membranes are reported to possess inherently low immunogenicity, further studies profiling acute and chronic immune responses, alongside strategies such as membrane “stealth” modifications, are crucial. Finally, our system distinguishes itself from other emerging anti-inflammatory nanotherapies by integrating two distinct, synergistic mechanisms: a biological membrane that actively targets inflammation and scavenges cytokines, coupled with the targeted intracellular delivery of a proven therapeutic agent (colchicine). This action approach contrasts with strategies relying solely on passive drug delivery or inert carrier materials, potentially offering a more comprehensive and potent means to quench the multifaceted inflammatory cascade post-MI. Future work will focus on comprehensive *in vivo* safety profiling, dose optimization studies, and comparative efficacy assessments against other nanoplatforms in advanced disease models.

### MM-LPs nanovesicles demonstrate favorable biosafety

**FIGURE 7:**
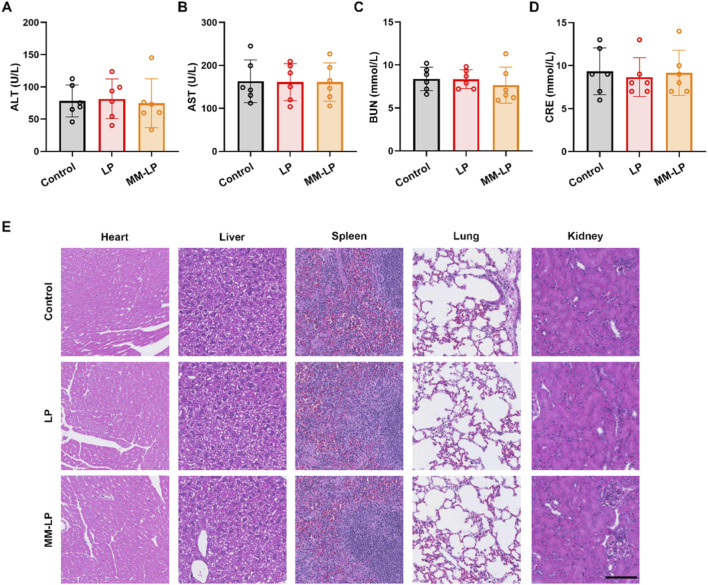
MM-LPs nanovesicles demonstrate favorable biosafety. **(A–D)** Biochemical markers relevant to hepatic and kidney function. Data are presented as mean ± SD (n = 6 per group). **(E)** Hematoxylin and eosin (H&E) staining of key organs from mice treated with LP nanoparticles and MM-LP nanoparticles, scale bar, 100 μm.

Given the enhanced therapeutic efficacy of the MM-LP nanovesicles established in this study, we next evaluated their biosafety. Clinical biochemical analysis confirmed that neither the LP nor MM-LP treatments induced detectable hepatic or renal toxicity. This was supported by serum levels of key markers, including alanine aminotransferase (ALT), aspartate aminotransferase (AST), blood urea nitrogen (BUN), and creatinine (CRE), all of which remained within normal limits following treatment (Figure 7A–D). H&E staining of pathological sections from multiple major organs was conducted to evaluate tissue compatibility. Comparative analysis revealed no significant histopathological alterations between the control group and the various treatment groups in MI mice (Figure 7E). These results indicate that both LP and MM-LP nanovesicles exhibit high biocompatibility following long-term administration in the MI model.

## Conclusion

The present study demonstrates that the macrophage membrane-based nanovesicle system (MM-LP@COL) effectively improves post-infarction outcomes through comprehensive anti-inflammatory mechanisms. The nanovesicles exhibited potent cytokine-neutralizing capacity against multiple pro-inflammatory mediators both *in vitro* and *in vivo*. More significantly, MM-LP@COL treatment substantially suppressed pro-inflammatory neutrophil recruitment across multiple compartments, including peripheral blood, bone marrow, and spleen, thereby disrupting the neutrophilic inflammatory cascade. This coordinated inhibition of both cytokine storm and neutrophil-driven inflammation contributed to preserved cardiac function, diminished myocardial inflammation, attenuated pathological remodeling, and ultimately an improved long-term prognosis after myocardial infarction. These findings position MM-LP@COL as a promising targeted strategy for controlling post-infarction inflammatory injury.

## Methods

### Macrophage membrane isolation

Raw 264.7 macrophage membranes were isolated using a hypotonic lysis method. Harvested cells were resuspended in a hypotonic lysate and subjected to four to five freeze-thaw cycles. The cell lysate was centrifuged at 850 *g* for 10 min at 4 °C. The supernatant was then collected and centrifuged at 15,000 × g for 30 min to obtain the membrane pellet. The membrane protein concentration was determined using a Pierce BCA protein assay kit (Thermo Fisher).

### Synthesis and characterization of MM-LP nanovesicles

MM-LP nanovesicles were prepared by the film hydration and extrusion method. A lipid film was formed from Soya PC, cholesterol, and DSPE-mPEG2000 in chloroform. The film was hydrated with PBS containing isolated macrophage membranes at a membrane protein-to-total lipid mass ratio of 1:5, followed by stirring at 500 rpm for 30 min at room temperature to form MM-LP nanovesicles. Control liposomes (LP) were prepared by hydrating the lipid film with PBS only. The suspensions were sequentially extruded through 400 nm, 200 nm, and 100 nm polycarbonate membranes. For specific experiments, DiD or COL was added to the initial lipid mixture during film formation to prepare MM-LP@DiD or MM-LP@COL.

The hydrodynamic diameter, polydispersity index (PDI), and zeta potential were measured by dynamic light scattering (DLS). Morphology was examined by transmission electron microscopy (TEM). Membrane fusion was assessed via the FRET technique using DiO and Dil, using a fluorescence spectrometer to record the emission spectra.

### Drug loading and encapsulation efficiency

The COL NPs were measured using a spectrophotometer (TECAN, Switzerland) at a wavelength of 345 nm. The LE and EE were calculated as follows:

$$\text{LE}=\frac{m\left(\text{COL}\right)}{m\left(\text{MM}-\text{LP}\right)+m\left(\text{COL}\right)}\times 100\%$$

$$\text{EE}=\frac{m\left(\text{COL}\right)}{m\left(\text{added}\right)}\times 100\%$$

where m(COL) is the mass of colchicine loaded in the NPs, m (MM-LP) is the mass of MM-LP NPs in the formulation, and m (added) is the mass of colchicine added.

### Inflammatory cytokine neutralization assay

The cytokine-neutralizing capacity of the MM-LP nanovesicles was quantitatively assessed *in vitro*. Briefly, varying concentrations of the nanovesicles (0, 1, and 3 mg/mL) were incubated with defined concentrations of recombinant inflammatory cytokines—IL-6 (2000 pg/mL), TNF-α (370 pg/mL), and IFN-γ (880 pg/mL)—in PBS at 37 °C for 30 min. After incubation, the mixtures were centrifuged at 16,000 × g for 15 min to remove nanovesicles. The supernatant was collected, and the concentrations of remaining free cytokines were measured by enzyme-linked immunosorbent assay (ELISA).

### Methods of animal experimentation

All animal procedures were conducted in accordance with the National Institutes of Health Guidelines for the Care and Use of Laboratory Animals and were approved by the Animal Ethics Committee of Wenzhou Medical University (Approval No. WYYYAEC-2021-235).

Male C57BL/6J mice (8 weeks old) were obtained from Beijing Vital River Laboratory Animal Technology Co., Ltd. The animals were housed under specific-pathogen-free (SPF) conditions with controlled temperature and humidity, a 12/12-h light/dark cycle, and were provided standard rodent diet and water *ad libitum*.

The myocardial infarction (MI) model was established through permanent ligation of the left anterior descending coronary artery (LAD). Briefly, mice were anesthetized with 2% isoflurane and anesthesia was maintained throughout the procedure. After thoracotomy, the LAD was ligated using a 7-0 silk suture. Body temperature was maintained throughout the procedure using a heated surgical platform.

Postoperatively, the mice were randomly allocated into four experimental groups: (1) PBS, (2) Free COL, (3) LP@COL, and (4) MM-LP@COL. Treatments were administered via tail vein injection according to a predefined dosing schedule. Cardiac function was assessed by echocardiography at predetermined time points, and all animals were humanely euthanized under deep anesthesia with pentobarbital (70 mg/kg, *ip*.) at the study endpoint.

### Enzyme-linked immunosorbent assay (ELISA)

Serum samples were collected after centrifugation at 3,000 *g* for 10 min at 4 °C and stored at −80 °C until analysis. Concentrations of inflammatory cytokines were measured using commercial ELISA kits according to the manufacturers’ instructions. Specifically, mouse TNF-α, IL-1β, and IL-6 levels were quantified using kits purchased from Lunchangshuo Biotech (Xiamen, China), while CCL2 concentrations were measured with a kit from Fankew (Shanghai, China).

During the assay procedure, standards and appropriately diluted serum samples were introduced into antibody-coated plates and incubated for 90 min at 37 °C. After washing to remove unbound material, biotinylated detection antibodies were added and incubated for 60 min. Subsequently, horseradish peroxidase (HRP)-conjugated streptavidin was introduced and incubated for 30 min. Tetramethylbenzidine (TMB) substrate was then added, and the chromogenic reaction was stopped after 15 min with sulfuric acid. Absorbance was measured at 450 nm, and cytokine concentrations were calculated by interpolation from the standard curve. All samples were assayed in duplicate to ensure reproducibility.

### Flow cytometric analysis

Single-cell suspensions were prepared from peripheral blood, spleen, and bone marrow for immunophenotyping based on established protocols as previously described (Sreejit et al., 2020; Yu et al., 2024). Peripheral blood was collected in EDTA-coated tubes and treated with erythrocyte lysis buffer (150 mM NH_4_Cl, 10 mM KHCO_3_, 0.1 mM EDTA) for 15 min at room temperature, followed by two washes with PBS. Spleens were gently dissociated through a 70 μm cell strainer, and the resulting cell suspension was similarly subjected to erythrocyte lysis and washed twice with PBS. Bone marrow cells were flushed from femurs using ice-cold PBS and passed through a 40 μm cell strainer.

Cell suspensions were first incubated with 7-AAD viability dye for 10 min to exclude non-viable cells from analysis. Subsequent surface marker staining was performed using fluorochrome-conjugated antibodies against the following targets: CD45, Gr-1, and CD115 for identification of neutrophils and monocytes; CD45, CD11b, CD19, and CD3 for discrimination of B and T lymphocytes; and CD4 and CD8 for T cell subset characterization. All antibody incubations were carried out at 4 °C in the dark for 40 min, followed by two washes with FACS buffer (PBS containing 2% fetal bovine serum) (for gating strategy, please see Supplementary Figure 3).

Data were acquired on a FACS Aria II flow cytometer (BD Biosciences) operated by FACSDiva software (v8.0.1). Compensation was calculated using single-stained controls, and fluorescence-minus-one (FMO) controls were included to guide accurate gating. For high-dimensional analysis, uniform manifold approximation and projection (UMAP) was applied to project cells into two dimensions based on their marker expression profiles. This approach successfully resolved distinct immune cell populations according to their lineage-defining markers.

### *In vivo* fluorescence imaging

To directly compare the cardiac targeting ability of the nanovesicles, DiD-labeled MM-LP and LP vesicles were prepared. Mice were subjected to myocardial infarction and, after 3 days, received a tail vein injection of either MM-LP@DiD or LP@DiD. At 3 days post-injection, mice were euthanized, and hearts were harvested for *ex vivo* fluorescence imaging to quantify the cardiac accumulation of the nanovesicles.

### Echocardiographic assessment

Cardiac function was evaluated in mice using high-resolution ultrasound imaging. Prior to examination, the thoracic hair was removed, and animals were anesthetized via inhalation of 1%–2% isoflurane. The anesthetized mice were positioned in supine posture on a temperature-controlled platform for physiological monitoring. Imaging was performed utilizing the Vevo high-resolution imaging system. Parasternal short-axis B-mode images were acquired at the left ventricular mid-papillary level, and two-dimensional measurements were taken to assess anterior and posterior wall thickness along with left ventricular internal diameter during end-diastole and end-systole.

### Histological analysis

Cardiac tissues were collected at the study endpoint for histopathological examination. After collection, tissues were rinsed with phosphate-buffered saline (PBS) and fixed in 4% paraformaldehyde for 24 h. The fixed specimens were then dehydrated through a graded ethanol series, embedded in paraffin, and sectioned into 4-μm slices using a microtome.

For histological assessment, hematoxylin and eosin (H&E) staining was first conducted to evaluate general tissue architecture and inflammatory cell infiltration. Wheat germ agglutinin (WGA) staining was then performed to outline cardiomyocyte membranes for subsequent cross-sectional area measurements. All stained sections were digitized with a slide scanner, and quantitative morphometric analyses of cardiomyocyte dimensions were carried out using ImageJ software with appropriate calibration.

### Statistical analysis

All data are presented as mean ± SD. Statistical significance was determined using one-way ANOVA followed by Tukey’s *post hoc* test. A *p*-value <0.05 was considered statistically significant. All statistical analyses were performed using GraphPad Prism version 8.

## Data Availability

The original contributions presented in the study are included in the article/Supplementary material, further inquiries can be directed to the corresponding authors.

## References

[B1] Adler Y. Charron P. Imazio M. Badano L. Barón-Esquivias G. Bogaert J. (2015). 2015 ESC guidelines for the diagnosis and management of pericardial diseases: the task force for the diagnosis and management of pericardial diseases of the european society of cardiology (ESC)Endorsed by: the european association for cardio-thoracic surgery (EACTS). Eur. Heart J. 36 (42), 2921–2964. 10.1093/eurheartj/ehv318 26320112 PMC7539677

[B2] Bai X. Guo Y. R. Zhao Z. M. Li X. Y. Dai D. Q. Zhang J. K. (2025). Macrophage polarization in cancer and beyond: from inflammatory signaling pathways to potential therapeutic strategies. Cancer Lett. 625, 217772. 10.1016/j.canlet.2025.217772 40324582

[B3] Bouabdallaoui N. Tardif J. C. Waters D. D. Pinto F. J. Maggioni A. P. Diaz R. (2020). Time-to-treatment initiation of colchicine and cardiovascular outcomes after myocardial infarction in the colchicine cardiovascular outcomes trial (COLCOT). Eur. Heart J. 41 (42), 4092–4099. 10.1093/eurheartj/ehaa659 32860034 PMC7700755

[B4] Cao W. Pham H. P. Williams L. A. Mcdaniel J. Siniard R. C. Lorenz R. G. (2016). Human neutrophil peptides and complement factor Bb in pathogenesis of acquired thrombotic thrombocytopenic purpura. Haematologica 101 (11), 1319–1326. 10.3324/haematol.2016.149021 27662014 PMC5394873

[B5] Deberge M. Yeap X. Y. Dehn S. Zhang S. Grigoryeva L. Misener S. (2017). MerTK cleavage on resident cardiac macrophages compromises repair after myocardial ischemia reperfusion injury. Circ. Res. 121 (8), 930–940. 10.1161/CIRCRESAHA.117.311327 28851810 PMC5623080

[B6] Finkelstein Y. Aks S. E. Hutson J. R. Juurlink D. N. Nguyen P. Dubnov-Raz G. (2010). Colchicine poisoning: the dark side of an ancient drug. Clin. Toxicol. (Phila) 48 (5), 407–414. 10.3109/15563650.2010.495348 20586571

[B7] Frangogiannis N. G. (2014). The inflammatory response in myocardial injury, repair, and remodelling. Nat. Rev. Cardiol. 11 (5), 255–265. 10.1038/nrcardio.2014.28 24663091 PMC4407144

[B8] Frangogiannis N. G. Smith C. W. Entman M. L. (2002). The inflammatory response in myocardial infarction. Cardiovasc Res. 53 (1), 31–47. 10.1016/s0008-6363(01)00434-5 11744011

[B9] Fung G. Luo H. Qiu Y. Yang D. Mcmanus B. (2016). Myocarditis. Circ. Res. 118 (3), 496–514. 10.1161/CIRCRESAHA.115.306573 26846643

[B10] Gao Y. Rong L. Cui J. Cheng W. Fu L. Fan G. (2025). Artificial lipids and macrophage membranes coassembled biomimetic nanovesicles for thoracic aortic dissection treatment. J. Control Release 383, 113844. 10.1016/j.jconrel.2025.113844 40379216

[B11] Jung M. Ma Y. Iyer R. P. Deleon-Pennell K. Y. Yabluchanskiy A. Garrett M. R. (2017). IL-10 improves cardiac remodeling after myocardial infarction by stimulating M2 macrophage polarization and fibroblast activation. Basic Res. Cardiol. 112 (3), 33. 10.1007/s00395-017-0622-5 28439731 PMC5575998

[B12] Li Y. Zhang Y. Lu J. Yin Y. Xie J. Xu B. (2021). Anti-inflammatory mechanisms and research progress of colchicine in atherosclerotic therapy. J. Cell Mol. Med. 25 (17), 8087–8094. 10.1111/jcmm.16798 34312998 PMC8419170

[B13] Li Y. Che J. Chang L. Guo M. Bao X. Mu D. (2022). CD47-and integrin α4/β1-Comodified-Macrophage-Membrane-Coated nanoparticles enable delivery of colchicine to atherosclerotic plaque. Adv. Healthc. Mater 11 (4), e2101788. 10.1002/adhm.202101788 34786845

[B14] Li Y. Wang J. Xie J. (2023). Biomimetic nanoparticles targeting atherosclerosis for diagnosis and therapy. Smart Med. 2 (3), e20230015. 10.1002/SMMD.20230015 39188346 PMC11236035

[B15] Li Y. Yu J. Wang Y. (2024). Mechanism of coronary microcirculation obstruction after acute myocardial infarction and cardioprotective strategies. Rev. Cardiovasc Med. 25 (10), 367. 10.31083/j.rcm2510367 39484142 PMC11522835

[B16] Lusis A. J. (2000). Atherosclerosis. Nature 407 (6801), 233–241. 10.1038/35025203 11001066 PMC2826222

[B17] Misawa T. Takahama M. Kozaki T. Lee H. Zou J. Saitoh T. (2013). Microtubule-driven spatial arrangement of mitochondria promotes activation of the NLRP3 inflammasome. Nat. Immunol. 14 (5), 454–460. 10.1038/ni.2550 23502856

[B18] Nahrendorf M. Pittet M. J. Swirski F. K. (2010). Monocytes: protagonists of infarct inflammation and repair after myocardial infarction. Circulation 121 (22), 2437–2445. 10.1161/CIRCULATIONAHA.109.916346 20530020 PMC2892474

[B19] Nidorf S. M. Fiolet A. T. L. Mosterd A. Eikelboom J. W. Schut A. Opstal T. S. J. (2020). Colchicine in patients with chronic coronary disease. N. Engl. J. Med. 383 (19), 1838–1847. 10.1056/NEJMoa2021372 32865380

[B20] Robertson S. Martínez G. J. Payet C. A. Barraclough J. Y. Celermajer D. S. Bursill C. (2016). Colchicine therapy in acute coronary syndrome patients acts on caspase-1 to suppress NLRP3 inflammasome monocyte activation. Clin. Sci. (Lond) 130 (14), 1237–1246. 10.1042/CS20160090 27129183

[B21] Slobodnick A. Shah B. Krasnokutsky S. Pillinger M. H. (2018). Update on colchicine, 2017. Rheumatol. (Oxford) 57 (Suppl. l_1), i4–i11. 10.1093/rheumatology/kex453 29272515 PMC5850858

[B22] Sreejit G. Abdel-Latif A. Athmanathan B. Annabathula R. Dhyani A. Noothi S. K. (2020). Neutrophil-derived S100A8/A9 amplify granulopoiesis after myocardial infarction. Circulation 141 (13), 1080–1094. 10.1161/CIRCULATIONAHA.119.043833 31941367 PMC7122461

[B23] Sun X. Duan J. Gong C. Feng Y. Hu J. Gu R. (2022). Colchicine ameliorates dilated cardiomyopathy *via* SIRT2-Mediated suppression of NLRP3 inflammasome activation. J. Am. Heart Assoc. 11 (13), e025266. 10.1161/JAHA.122.025266 35766262 PMC9333380

[B24] Tardif J. C. Kouz S. Waters D. D. Bertrand O. F. Diaz R. Maggioni A. P. (2019). Efficacy and safety of low-dose colchicine after myocardial infarction. N. Engl. J. Med. 381 (26), 2497–2505. 10.1056/NEJMoa1912388 31733140

[B25] Van Echteld I. Wechalekar M. D. Schlesinger N. Buchbinder R. Aletaha D. (2014). Colchicine for acute gout. Cochrane Database Syst. Rev. (8), Cd006190. 10.1002/14651858.CD006190.pub2 25123076

[B26] Van Wagoner D. R. (2011). Colchicine for the prevention of postoperative atrial fibrillation: a new indication for a very old drug? Circulation 124 (21), 2281–2282. 10.1161/CIRCULATIONAHA.111.057075 22105193 PMC3256985

[B27] Veillette A. Chen J. (2018). SIRPα-CD47 immune checkpoint blockade in anticancer therapy. Trends Immunol. 39 (3), 173–184. 10.1016/j.it.2017.12.005 29336991

[B28] Wang Y. Dembowsky K. Chevalier E. Stüve P. Korf-Klingebiel M. Lochner M. (2019). C-X-C motif chemokine receptor 4 blockade promotes tissue repair after myocardial infarction by enhancing regulatory T cell mobilization and immune-regulatory function. Circulation 139 (15), 1798–1812. 10.1161/CIRCULATIONAHA.118.036053 30696265 PMC6467561

[B29] Yu J. Li Y. Hu J. Wang Y. (2024). Interleukin-33 induces angiogenesis after myocardial infarction via AKT/eNOS signaling pathway. Int. Immunopharmacol. 143 (Pt 3), 113433. 10.1016/j.intimp.2024.113433 39486188

[B30] Zhang T. Zhang Y. Cui M. Jin L. Wang Y. Lv F. (2016). CaMKII is a RIP3 substrate mediating ischemia- and oxidative stress-induced myocardial necroptosis. Nat. Med. 22 (2), 175–182. 10.1038/nm.4017 26726877

