# Supplementary material for: A biohybrid nanovesicle hijacks inflammatory chemotaxis to deliver colchicine for myocardial infarction therapy
Source: Front Bioeng Biotechnol. 2026 Apr 2;14:1751640. doi: 10.3389/fbioe.2026.1751640 (PMC13083181; doi:10.3389/fbioe.2026.1751640)
Supplement: Supplementary file 1 [file Supplementaryfile1.docx]

**Supplementary Figures**

**
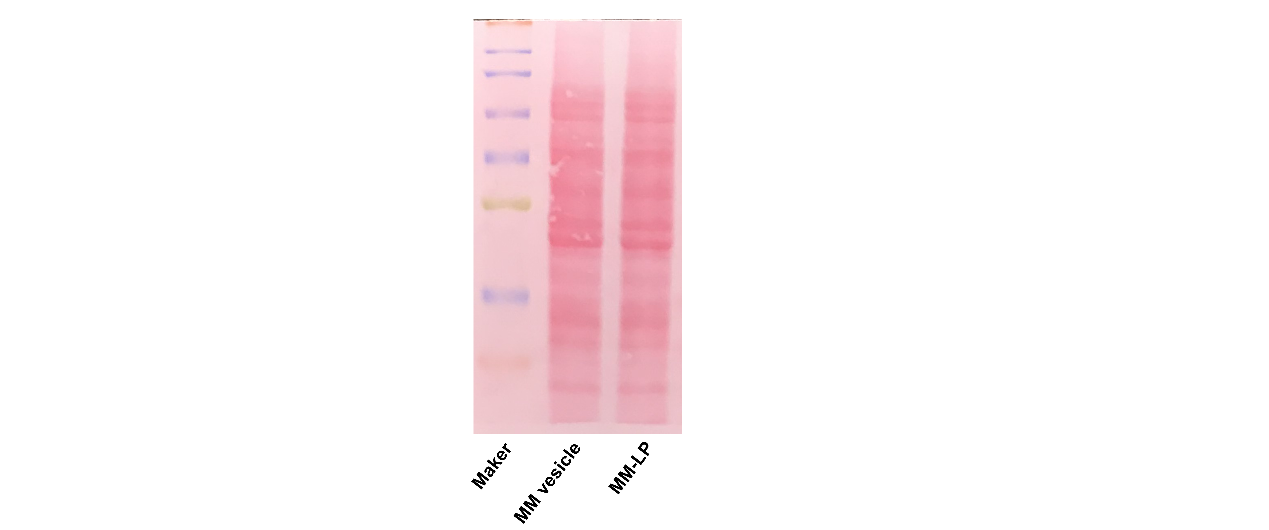
 Supplementary Figure 1. The protein profiles of the MM vesicle and MM-LP determined by Ponceau S.**

**
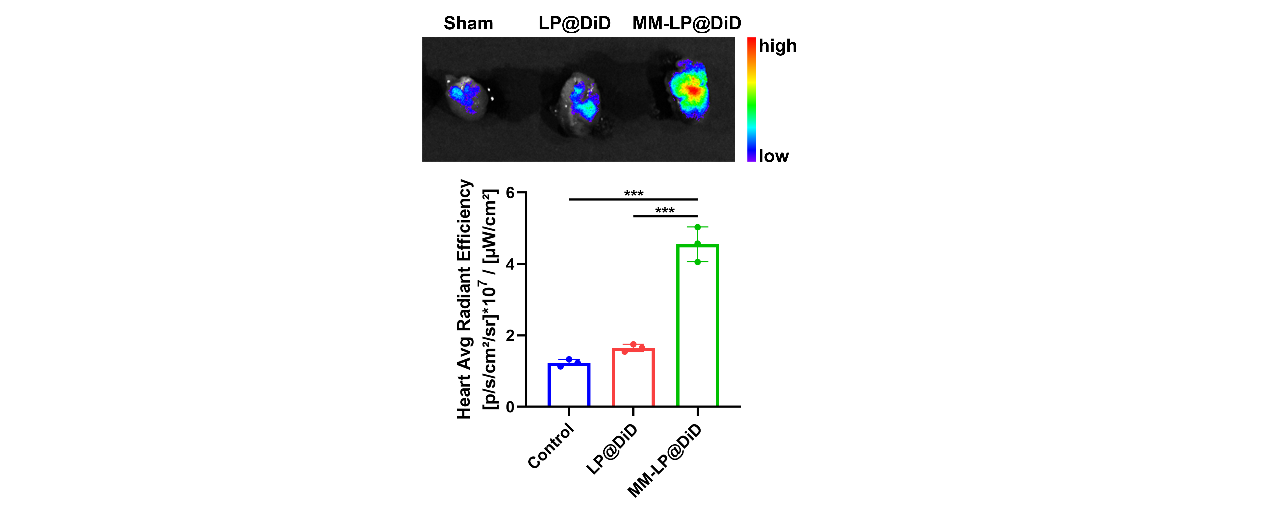
**

**Supplementary Figure 2. Representative *ex vivo* fluorescence images of DiD fluorescence dye accumulated in heart at 24 h postintravenous injection of LP@DiD and MM-LP@DiD.**

**
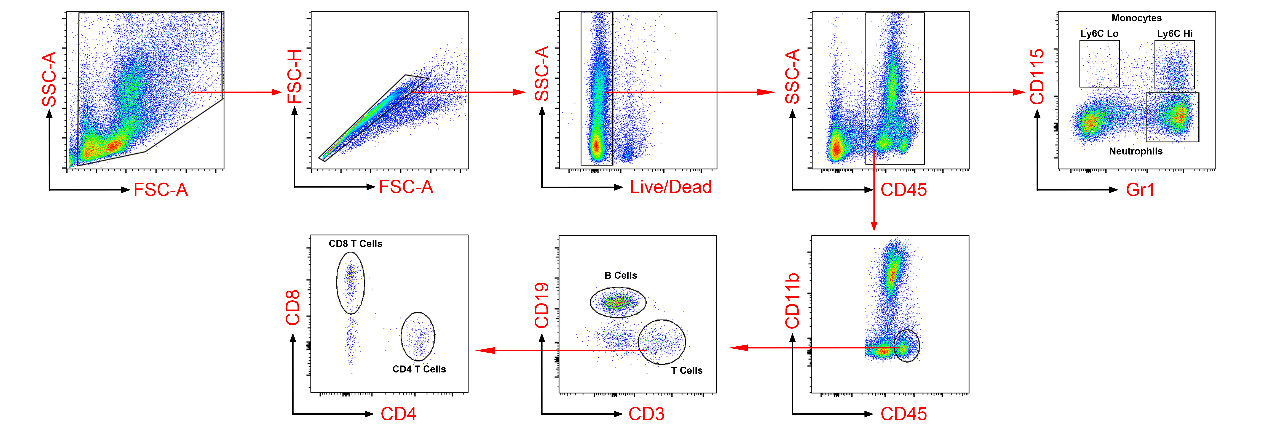
**

**Supplementary Figure 3. Gating strategy for identification of leukocytes in mouse.**
